# Adverse events associated with robotic-assistance in total hip arthroplasty: an analysis based on the FDA MAUDE database

**DOI:** 10.1177/11207000241263315

**Published:** 2024-08-04

**Authors:** S Bradley Graefe, Gregory J Kirchner, Natalie K Pahapill, Hannah H Nam, Mark L Dunleavy, Nikkole Haines

**Affiliations:** Department of Orthopaedics and Rehabilitation, Penn State College of Medicine, Hershey, PA, USA

**Keywords:** Complications, robot, robotic-assisted, surveillance, total hip arthroplasty

## Abstract

**Background:**

The Food and Drug Administration (FDA) maintains the Manufacturer and User Facility Device Experience (MAUDE) database for reporting adverse events associated with medical devices, including emerging technologies, such as robotic-assisted total hip arthroplasty (THA). Aim of this study was to evaluate the variation of adverse events associated with robotics in THA.

**Methods:**

Medical device reports (MDRs) within the MAUDE database were identified between 2017 and 2021. For MDR identification the product class “orthopaedic stereotaxic equipment” and terms associated with THA were used. Individual adverse events were identified and organised by type and consequences, such as patient injury, surgical delay, or conversion to the manual technique.

**Results:**

521 MDRs constituting 546 discrete events were found. The most common reported complication was intraoperative hardware failure (304/546, 55.7%), among which the most common failure was a broken impaction handle/platform (110, 20.1%). Inaccurate cup placement was the second most common reported complication (63, 11.5%). Abandoning the robot occurred in 13.0% (71/521) of reports. A surgical delay was noted in 28% (146/521) of reports, with an average delay of 17.9 (range 1–60) minutes.

**Conclusions:**

Identifying complications that may occur with robotics in THA is an important first step in preventing adverse events and surgical delays. Database analysis provide an overview of the range of complications.

## Introduction

Total hip arthroplasty (THA) is 1 of the most frequently performed orthopaedic procedures, with patient satisfaction rates often reported at 90% or higher.^1,2^ Despite this, the number of patients undergoing revision THA continues to rise and is most frequently as a result of dislocation or mechanical loosening.^3^ In an attempt to mitigate these postoperative complications, robotic-assisted THA has been developed and demonstrated improved accuracy of component placement and mechanical alignment when compared to manual techniques.^4–6^ As our understanding of the hip-spine relationship increases, the advantages of more specific acetabular cup positioning have been advocated to minimise the burden of instability and revisions, especially in patients with spinopelvic pathologies.^7^ The usefulness of navigation and robotics in determining the optimal placement and orientation in this patient population has been debated; however, robotic-assisted THA has been proposed as a means to assist surgeons with achieving this goal. But no studies have demonstrated associated improvements in revision rates, functional outcomes, or implant survivorship for robotic-assisted THA.^8,9^ Nevertheless, the utility of robotic-assisted THA has been projected to overtake manual techniques by 2028 and account for nearly 70% of all THAs by 2030.^10,11^

Robotic-assisted THA was first introduced in the 1990s and continues to evolve.^12^ Systems are now categorised as “fully active” if the system functions independently of the surgeon, versus “semi-active” if the system provides guidance while still enabling the surgeon to maintain overall control. Although systems vary, a computed tomography (CT) scan is generally obtained preoperatively and imported into the robotic software. This is then used to calculate implant sizes and positioning, as well as acetabular bone reaming depth and the femoral osteotomy site. Most systems require placement of either pins or screws into the iliac crest and proximal femur to allow software registration of anatomic landmarks. An interactive robotic arm is then utilised to perform acetabular preparation and implant placement.^6,12^ With the additional complexity, including other surgical steps and instrumentation, there is added potential for intra- and postoperative adverse events. New complications such as retained foreign bodies from fractured checkpoint pins, pin site fractures, pin site infections, software malfunctions, and mechanical failures of the robotic platform are possible.^13^ It is imperative to identify and document all possible complications, so both surgeons and patients can make educated decisions when choosing between robotic-assisted and manual techniques for THA.

In this study, we identified and categorised adverse events related to robotic-assisted THA by utilising the Manufacturer and User Facility Device Experience (MAUDE) database from the United States Food and Drug Administration (FDA). Pagani et al.^13^ previously utilised this database to assess complications related to robotic-assisted arthroplasty, however this study primarily reported total and partial knee arthroplasty data and was limited by a low number of analysed event reports related to robotic-assisted THA. Therefore, the goal of this study was to evaluate the MAUDE database for adverse events associated with contemporary robotic-assisted THA systems in a larger scale and provide a review of the range of complications that can occur. Knowledge of the possible adverse events associated with robotic-assisted THA may assist in preventing patient injury and minimise surgical delays.

## Materials and methods

The MAUDE database is maintained by the FDA and it contains medical device reports (MDRs), which are descriptions of incidents where a medical device may have directly or indirectly contributed to patient injury or death.^14^ FDA mandated reporters submit MDRs to the MAUDE database. These include device manufacturers or importers, as well as the facilities where devices are used. MDRs may also be submitted by voluntary reporters, which include health care professionals or patients. The MAUDE database includes publicly accessible MDRs since at 1993. MDRs contain de-identified event descriptions and are compliant with the Health Insurance Portability and Accountability Act (HIPAA).^14^ Due to the de-identified, this study was exempt from review by the corresponding institutional review board.

**Figure 1.**
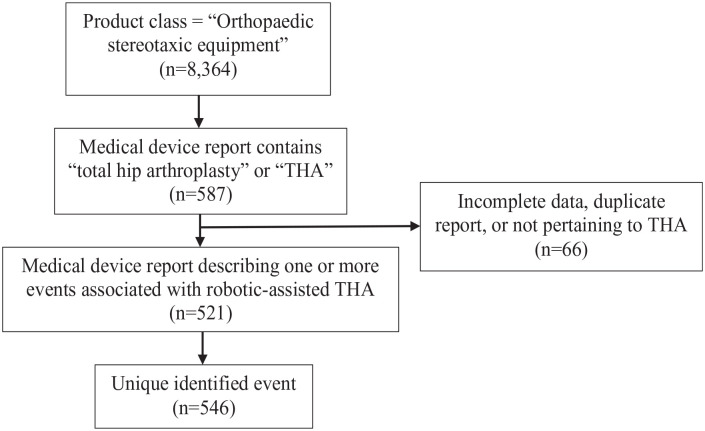
Flow diagram of identifying medical device reports and adverse events associated with robotic-assisted THA within the FDA MAUDE database.

The MAUDE database assigns a product class to all devices. Robotic devices are designated under the product class “orthopaedic stereotaxic equipment.” 8364 unique MDRs involving the product class “orthopaedic stereotaxic instrument” were identified during a 5-year period from 01 January 2017 to 31 December 2021. These MDRs were searched for terms “total hip arthroplasty” and “THA,” yielding in a total of 587 MDRs. Event reports unrelated to robotic-assisted THA, duplicate reports, and those with inadequate information were excluded (Figure 1).

Each MDR was independently analysed. For each report, data regarding event type, patient injury, case cancellation, need for operative reintervention, surgical delay, and conversion to manual arthroplasty were recorded. All events were categorised *posthoc* into 7 types: intraoperative hardware failure; inaccurate cup placement; inaccurate reaming; fracture; software failure; and miscellaneous. The proportion of event types contributing to surgical delay or conversion to manual THA were analysed.

## Results

A total of 521 MDRs were identified, which included descriptions of 546 discrete events (Table 1). Surgical delay was noted in 28.0% (146/521) of reports, with an average delay of 17.9 minutes (range 1–60 minutes). Conversion to manual technique was noted in 13.0% (71/521) of the reports, there were no cancelled cases. Patient injury was noted in 14.1% (77/521) of reports, and 7.3% (38/521) reports described a reintervention.

**Table 1.** Summary of medical device reports of robotic-assisted THA in the FDA’s MAUDE database from 2017 to 2021.

Total adverse event reports	521
Total adverse event report complications	546
Surgery delay reported, *n* (%)	146 (28.0)*
Average delay (minutes)	17.9
Range (minutes)	1–60
Conversion to manual, *n* (%)	71 (13.0)*
Case canceled, *n* (%)	0
Patient injury, *n* (%)	77 (14.1)*
Surgical reintervention, *n* (%)	38 (7.3)*

MAUDE, Manufacturer and User Facility Device Experience; FDA, US Food and Drug Administration; THA, total hip arthroplasty.

* Indicates 521 used as denominator.

Intraoperative hardware failure was the most common reported adverse event (304/546, 55.7%) (Table 2). The most common type of intraoperative hardware failure was a broken impaction handle or platform (110/546, 20.1%). Incorrect acetabular cup placement was the second most common reported complication (95/546, 17.4%). 63 cases (11.5%) of inaccurate acetabular cup placement were noticed intraoperatively and 32 cases (5.9%) postoperatively, respectively. Software failure was reported in 52 cases (9.5%). The most common cause of software failure was the inability to successfully complete bone registration (12/546, 2.2%), followed by camera connection issues (9/546, 1.6%), unresolvable error messages (8/546, 1.5%), and software not correlating with the patient’s anatomy (7/546, 1.3%).

**Table 2.** Types of events described in device reports of robotic-assisted THA in the FDA’s MAUDE database from 2017–2021 (*n* = 546).

**Intraoperative hardware failure**	304	55.7%
Broken impaction handle or platform	110	20.1%
Foreign body	55	10.1%
Broken array not otherwise specified	50	9.2%
Broken hip end effector	40	7.3%
Broken reamer	24	4.4%
Trigger jammed or broken	10	1.8%
Broken checkpoint, unspecified	9	1.6%
Broken articulating screwdriver	2	0.4%
Broken checkpoint driver	2	0.4%
Handpiece detached from reamer	1	0.2%
Acetabular cup broke during impaction	1	0.2%
**Inaccurate cup placement**	63	11.5%
Inaccurate anteversion or inclination noticed intraoperatively	32	5.9%
Inaccurate anteversion or inclination noticed postoperatively	20	3.7%
Cup impacted deeper in reality than displayed on software	7	1.3%
Cup more proud in reality than displayed on software	4	0.7%
**Software Failure**	52	9.5%
Unable to successfully complete bone registration	12	2.2%
Camera connection issues	9	1.6%
Unresolvable error message	8	1.5%
Software not correlating with anatomy	7	1.3%
Anteversion & retroversion flipped on software	5	0.9%
Black or frozen display	5	0.9%
Inaccurate measurements, not otherwise specified	4	0.7%
Cup Impaction software malfunction	1	0.2%
Index error reported during impaction	1	0.2%
**Postoperative complications**	52	9.5%
Periprosthetic fracture	14	2.6%
Postoperative aseptic loosening	11	2.0%
Leg-length discrepancy	10	1.8%
Postoperative dislocation	10	1.8%
Postoperative infection	6	1.1%
Postoperatively reported cobalt toxicity	1	0.2%
**Miscellaneous**	35	6.4%
Unexpected robot movement	12	2.2%
Black residue on equipment due to poor sanitation	5	0.9%
Power cord connection issues	5	0.9%
Presurgical planning performed on the non-operative side	4	0.7%
During reaming, torque from reamer twisted surgeon’s wrist	3	0.5%
Excessive noise from the robot during use	2	0.4%
Electrical complication/fire with robot	1	0.2%
Handpiece snapped backwards during reaming	1	0.2%
Recalled part	1	0.2%
Robot was unable to lock in haptic (resting) position	1	0.2%
**Inaccurate reaming**	32	5.9%
Reamer ran outside of haptics boundary	18	3.3%
Over reamed medial wall (acetabulum)	6	1.1%
Over reamed posterior wall (acetabulum)	5	0.9%
Inaccurate ream from pre-operative plan	3	0.5%
**Fracture**	8	1.5%
Acetabular fracture	5	0.9%
Greater trochanter fracture	3	0.5%

MAUDE, Manufacturer and User Facility Device Experience; FDA, US Food and Drug Administration; THA, total hip arthroplasty.

The most common causes for conversion to the manual technique were software failure (32/75, 42.7%) (Table 3), inaccurate reaming (12/75, 16.0%), and inaccurate cup placement (11/75, 14.7%). The most common cause of software failure resulting in conversion was an inability to complete bone registration (8/75, 10.7%) followed by camera connection issues (6/75, 8.0%).

**Table 3.** Types of events associated with conversion of robotic-assisted THA to manual THA (*n* = 75).

**Software failure**	32	42.7%
Unable to successfully complete bone registration	8	10.7%
Camera connection issues	6	8.0%
Unresolvable error message	6	8.0%
Software not correlating with anatomy	3	4.0%
Anteversion & retroversion flipped on software	2	2.7%
Black or frozen display	4	5.3%
Inaccurate measurements, not otherwise specified	2	2.7%
Cup Impaction software malfunction	1	1.3%
**Miscellaneous**	15	20.0%
Unexpected robot movement	8	10.7%
Presurgical planning performed on the non-operative side	1	1.3%
Power cord connection issues	1	1.3%
Leg Length discrepancy	1	1.3%
During reaming, torque from reamer twisted surgeon’s wrist	1	1.3%
Excessive noise from the robot during use	1	1.3%
Electrical complication/fire with robot	1	1.3%
**Inaccurate reaming**	12	16.0%
Reamer ran outside of haptics boundary	6	8.0%
Over reamed medial wall (acetabulum)	3	4.0%
Over reamed posterior wall (acetabulum)	2	2.7%
Inaccurate ream from pre-operative plan	1	1.3%
**Inaccurate cup placement**	11	14.7%
Inaccurate anteversion or inclination noticed intra-operatively	9	12.0%
Cup impacted deeper in reality than displayed on software	1	1.3%
Cup more proud in reality than displayed on software	1	1.3%
**Intraoperative hardware failure**	4	5.3%
Broken impaction handle or platform	1	1.3%
Broken reamer	1	1.3%
Trigger jammed or broken	2	2.7%
**Fracture**	1	1.3%
Acetabular fracture	1	1.3%

THA, total hip arthroplasty.

Intraoperative hardware failure was the most common reason for surgical delay (83/151, 55.0%), with an average surgical delay of 12.3 minutes (range 1–35 minutes) (Table 4). The second most common cause of a reported surgical delay was due to software failure (31/151, 20.5%), resulting in an average delay of 19.0 minutes (range 5–60 minutes). Inaccurate cup placement accounted for 9.3% (14/191) of reported delays but resulted in the longest average delay of 30.2 minutes (10–60 minutes).

**Table 4.** Types of events associated with surgical delay (*n* = 151).

	*n*	Percent	Average delay (min)	Range (min)
**Intraoperative hardware failure**	83	55.0%	12.3	1–35
Broken impaction handle	19	12.6%	15.3	5–35
Broken array not otherwise specified	16	10.6%	9.1	1–30
Broken reamer	16	10.6%	12.2	1–30
Foreign body from pelvic array	13	58.6%	12.3	5–15
Robotic handpiece malfunction	13	8.6%	11.3	2–30
Foreign body unspecified, removed	4	2.6%	16.3	15–20
Array sensory disconnection	2	1.3%	8.5	2–15
**Software failure**	31	20.5%	19.0	5–60
Unable to complete registration	6	4.0%	16.7	5–30
Camera connection issue	6	4.0%	24.2	5–30
Reamer running outside of haptics	5	3.3%	13.0	5–30
Unresolvable error message	4	2.6%	15.0	5–20
Software not matching anatomy	3	2.0%	31.7	5–60
Frozen screen	3	2.0%	20.0	10–30
Discrepancy between reamer software location	2	1.3%	25.0	5–45
Anteversion retroversion flipped	2	1.3%	7.5	5–10
**Inaccurate cup placement**	14	9.3%	30.2	10–60
Inaccurate anteversion and retroversion	9	6.0%	27.8	15–45
Cup too proud	3	2.0%	28.3	10–60
Inaccurate cup placement not otherwise specified	1	0.7%	30	30
Poor cup placement due to poor patient bone quality	1	0.7%	60	60
**Miscellaneous**	10	6.6%	15.5	5–30
Inadequate sterilisation	4	2.6%	12.5	5–15
Unexpected robotic movement	3	2.0%	10	10
Inaccurate pre-surgical planning (wrong side)	3	2.0%	25	15–30
**Inaccurate reaming**	10	6.6%	17.5	10–30
Over reamed posterior wall	5	3.3%	18.0	15–30
Over reamed medial wall	3	2.0%	18.3	10–30
Inaccurate reaming not otherwise specified	2	1.3%	15	15
**Fracture**	3	2.0%	30	15–60
Acetabular fracture	3	2.0%	30	15–60

min, minutes.

## Discussion

Robotic-assisted THA has been developed to improve surgeon accuracy of component placement and mechanical alignment compared to conventional surgical techniques.^4–6^ However, studies have not shown an association between robotic-assisted techniques and decreased revision rates, functional outcomes, or implant survivorship and these techniques may come with a unique profile of complications.^8,15^

The MAUDE database permits analysis of reported adverse events associated with robotic-assisted THA across a range of clinical settings. This database accepts reports from hospitals and practices nationwide and does not limit data from single, high-volume institutions.^14^ Over ¼ of reports described surgical delay, and over 10% described patient injury or abandoning the robotic system.

This study has several limitations. The database utilises a “passive surveillance system,” and events may be underreported.^16,17^ The FDA states that “the incidence or prevalence of an event cannot be determined” from the MAUDE database.^14^ For example, 1 observer may portray and report a software malfunction of the robot as an adverse event, while another observer may not. Additionally, specific content and level of detail illustrated in each event description is dependent on the individual submitting the MDR. Therefore, inconsistencies between MDRs exist due to individual reporting discrepancies. As a result, a few MDRs needed to be excluded from this study due to incomplete information presented in the report. Another limitation is the influence of patient-specific factors such as body habitus, anatomic complexity, comorbid conditions, and individual patient expectations, all of which may affect the ability to successfully implement robotics within hip arthroplasty. In addition, we did not compute additional costs and training required for surgeons and staff in this study. Lastly, it is important to account for the diversity in technical skill of each surgeon in using robotic techniques, and the MAUDE database does not control for surgeon experience or surgical setting (e.g., high-volume versus low-volume arthroplasty centres).

Pagani et al.^13^ previously described adverse events associated with robotic-assisted THA, using the MAUDE database too. The study included unicompartmental knee arthroplasty, total knee arthroplasty, and THA. They found that retained registration checkpoint hardware was most commonly reported adverse event in robotic-assisted THA, followed by discrepancy between actual and displayed reamer location, and acetabular medial wall violation. When cases converted to manual surgery, medial wall violation at the acetabulum and inability to accurately complete bone registration were leading causes for failure. However, this study only included 44 reports of robotic-assisted THA, and the study timeline, between January 2020 and July 2021, was potentially affected by the consequences of the COVID-19 pandemic.^13,18^

The goal of robotic-assisted THA includes improved component positioning and orientation through reduction of human error. This should lead to a reduced incidence of dislocation and impingement, and improve patient pain and functioning. Robotic-assisted THA is still relatively uncommon compared to surgical robotics in other fields.^19^ Previous studies have compared complication rates between robotic-assisted and conventional THA; however, the data is somewhat conflicting. In a meta-analysis comparing robotic-assisted THA to conventional THA across 7 studies and 1516 patients, Chen et al.^12^ found that there was no difference in surgical time and postoperative complication rates, although intraoperative complication rates and total complication rates were higher among robotic-assisted cases. Kunze et al.^15^ performed a systematic review of randomised control trials of robotic-assisted and computer-assisted THA and found that overall, robotic-assisted THA had a higher rate of acetabular cups placed in the Lewinnek safe zone but had longer surgical times and similar incidences of complications and revisions compared to conventional THA.^9^ A large retrospective cohort study found no differences in intraoperative or immediate postoperative complications between patients who underwent robotic-assisted or conventional THA.^20^ Our study demonstrates that the range of possible complications grows when a robotic system is utilised, which might not have been fully appreciated in previous comparisons of robotic-assisted versus conventional THA. It is important to note that we did not evaluate incidence or rates of complications for robotic hip replacements, or compare relative complication rates between conventional and robotic-assisted THA.

From 2000 to 2014, the annual THA incidence increased 105%, with growth rates projected to continue to increase through 2030.^21^ Despite hip arthroplasty being touted as the “operation of the century,” it has been shown that nearly 50% of aseptic hip revisions might be preventable.^22,23^ New technological advancements require significant training and expertise to establish proficiency. Therefore, the learning curve accompanying new robotic surgical technologies may contribute to rates of complications. A study that analysed the first 100 robotic THA surgeries by an orthopaedic surgeon experienced in conventional THA found that 14 procedures were required to overcome the learning curve.^24^ Another single-surgeon study found a learning curve of 35 cases was needed to reduce the risk of acetabular component outliers and reduce operative time.^25^ Therefore, adverse events may occur more frequently during early adoption. The data from our study can help surgeons become cognisant of possible complications that might occur with robotic THA and enable avoidance of these adverse events or preparedness if they do occur.

If new surgical technologies such as robotics are introduced, it is important to develop established standards in surgical training and adverse reporting guidelines to analyse, investigate, and reduce adverse events. Patient safety and benefit should always be prioritised when implementing innovative techniques; therefore, documentation and dispersion of adverse events is crucial. The findings from this study reveal qualitative information of adverse events to be considered by surgeons and manufacturers when introducing robot-assisted THA systems.
